# A GIS-based framework to assess heatwave vulnerability and impact scenarios in urban systems

**DOI:** 10.1038/s41598-023-39820-0

**Published:** 2023-08-11

**Authors:** Valeria D’Ambrosio, Ferdinando Di Martino, Vittorio Miraglia

**Affiliations:** 1 Dipartimento di Architettura, Università degli Studi di Napoli Federico II, Via Toledo 402, 80134 Naples, Italy; 2 Centro Interdipartimentale di Ricerca “A. Calza Bini”, Università degli Studi di Napoli Federico II, Via Toledo 402, 80134 Naples, Italy

**Keywords:** Climate change, Computer science, Information technology

## Abstract

In this work, we propose a GIS-based platform aimed at the analysis of heatwave scenarios risks produced in urbanised environments, applied to assess vulnerability and impact heatwave scenarios. Our framework implements a hierarchical model that represents a good trade-off between forecast accuracy and portability in different urban fabrics, apart from the spatial scale of the data, using topographic and remote sensing spatial data provided by institutional agencies. The framework has been applied to two study areas: the dense city of Naples (Italy) and the intermediately populated city of Avellino (Italy) in order to evaluate its accuracy performances and portability in different urban fabrics. Our framework can be used by urban planners and decision makers as a tool to locate potential risk zones where it is necessary to implement climate-resilient solutions.

## Introduction

The increasing need to address climate hazards in urban tissues encouraged researchers to develop decision-support tools to assess risk levels and identify critical urban areas where climate-resilient solutions must be adopted.

In recent years, some researchers have developed studies aimed at understanding the relationships between the built environment and climate phenomena in order to define adaptation strategies and programs that aim to reduce the risks caused by climate change. These studies have resulted in the development of methodologies, procedures, guidelines, web services, and tools to increase awareness of climate vulnerabilities and risks and support the development of local adaptation strategies and plans^1–7^.

Many of these contributions, in particular those focused on assessing the climate vulnerability of the urban system, base the starting analysis on a synthesized set of characteristics of the study applying statistical methods and approaches, such as the Principal Component Analysis (PCA).

PCA is a consolidated multivariate statistical method aimed at aggregating information and reducing the number of variables; it is used to assess the spatial distribution of the vulnerability indicator based on a reduced set of characteristics of the urban model. However, although this method is used in various studies that estimate the heatwave vulnerability of urban systems, it has limitations and shortcomings mainly due to poor model portability and usability in different urban contexts and on different scales. Its limitation is the inability to identify peculiarities specific to the study's geographical context; this implies that the same characteristics may not be attributable to urban systems located in different territorial contexts.

Wolf and McGregor^8^ create a heatwave vulnerability index for a region stretching over Greater London by census area by using the PCA approach to represent a collection of relevant characteristics. In our opinion, due to the identification of traits that are intimately tied to the territorial environment, the procedure cannot be replicated in a different urban design.

A study of the impact of the heatwave on urban patterns is proposed in^9^; the authors apply their method to the city of Paris, taking into consideration five scenarios of urban expansion over time, depending on the climatic conditions. The results of the study show that in urban areas with a high concentration of inhabitants the impact of the phenomenon is greater.

In^4^ ana analysis of the relationship between heatwave vulnerability and mortality is carried out in the New York City study area. The results show that mortality during heatwave periods is higher in zones with the highest mean surface temperature and lower in areas with the highest density of green spaces.

In^10^ the authors propose a study conducted in fifteen urban patterns in Europe and the United States aimed at assessing the heatwave vulnerability of urban fabrics. This study highlights that the spatial distributions of vulnerability in these urban patterns are different from each other, showing that different urban fabrics are in relation to different local situations. A local study is therefore necessary to evaluate which are the specific characteristics of an urban pattern that affect heatwave vulnerability.

In^3^ the PCA algorithm is applied to eight variables that affect the heatwave vulnerability of an urban pattern. The experimentation is carried out in a study area that includes the city of Osaka in Japan. The eight variables are extracted from population data by census area and from land use data. The variables that have the greatest impact on vulnerability are the age of the population, the education and social condition of the population, and the density and size of green areas.

Several additional research studies^11–14^ support the continued evolution of the vulnerability concept, which places a strong emphasis on local assessment to better understand how the vulnerability features are connected to losses and damages during and after the occurrence of climatic events.

A critical point of these models is the use of local data at a detailed scale, which limits their portability on different urban models; in addition, the use of too many parameters makes these models unsuitable to be applied in different contexts.

To overcome these limitations, we propose a framework implemented in a GIS environment that can ensure both the portability and reusability of the model for different urban patterns; to achieve this goal, we propose the use of a hierarchical model for assessing vulnerability and heatwave impacts on urban systems that does not require a large number of parameters and can be replicated on different urban fabrics. Furthermore, the accuracy of vulnerability and impact assessments.

By replicable model, we suggest a vulnerability and impact assessment model that incorporates measurable parameters through the collection of data available and tested by institutional bodies and available in different urban fabrics. Coming from different institutional sources, these datasets are not homogeneous, and a reconciliation activity was necessary to bring them back to the same coordinate system and reproduce the correct coding of the fields to be able to acquire and use them in the algorithms developed in a GIS platform for the generation of impact scenarios.

For this purpose, the census section is considered as the atomic space unit, representing a unit recognized as homogenous for urban characteristics and on which the censuses of the characteristics of the population, buildings, etc. are carried out by the institutional bodies in charge. This choice represents a trade-off between the need to use information on a large scale, which allows greater accuracy in estimates but reduces the portability of the model, and the use of too small scale of data, which could affect the accuracy of the results.

Our framework is based on the model proposed in^15^ to assess vulnerability and heatwave impact scenarios on urban systems. The decision to use this model is mainly linked to the hierarchical structure adopted to subdivide the urban system, to the use of a small number of parameters that are easily measurable at the spatial scale of the census sections, and above all to its accuracy, which was measured in the experimentation carried out in the METROPOLIS research project^15^.

This model was tested on two districts in the east and west of the city of Naples (Italy). It breaks down the system into three subsystems: the residential buildings, the open spaces, and the residential population. The model assesses the vulnerability of each subsystem and uses the IPCC AR5 model described in^16^ to evaluate the impacts generated by heatwave hazard scenarios. We use this model to obtain subsystems vulnerability estimates and of the impact scenarios attributed to the individual census areas.

Our research was conducted as part of the PLANNER project^17,18^, in which a geocomputational platform was tested, breaking down the urban system into atomic units made up of the individual census sections. In order to evaluate the performances and the portability of our framework, it was developed and tested on two study areas: the city of Naples, a complex urban system with a very high build density, and the city of Avellino, a less dense context both for building and housing density.

The prerequisite for making our framework portable is the choice to consider the census section as an atomic spatial entity. The primary factor is the census section's ability to reflect the smallest spatial unit with uniformly homogenous urban and territorial features. For this reason, the indicators used for evaluating the vulnerability and impacts of the heatwave phenomenon have been aggregated for census sections.

In the following section, we introduce the model used for our studies to better explain the meaning of the proposal; it is possible to read about the structure of the hierarchical model and how it was built. In the third section, we present our framework, and in "Results and discussion" section are shown the results of the application of our framework on two different urban fabrics given by the cities of Naples and Avellino. The conclusions about our work are given in "Conclusions" section.

## Preliminaries

### The hierarchical vulnerability and impact model

The model chosen to develop our framework is the one defined in^15^ for the generation of the impact scenarios; it is based on the general model proposed in the AR5 report of the IPPC—Intergovernmental Panel on Climate Change^16^, which assesses the impacts of climatic phenomena as a result of the combination of vulnerability, hazard and exposure.

An impact scenario is generated by referring to a specific hazard scenario caused by a climatic phenomenon that acts on an urban system whose vulnerability to the phenomenon has been assessed. The AR5 report evaluates the effects of climate change by taking into account the subsystems' vulnerabilities whereby the exposure interacts.

**Figure 1:**
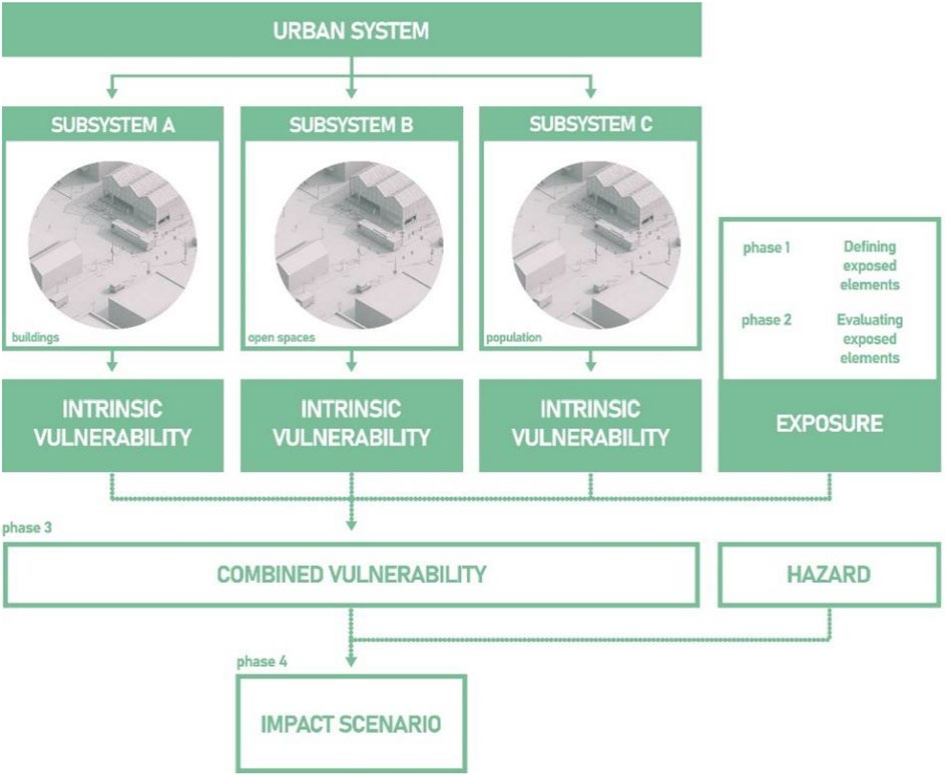
Scheme of the process applied in^15^ to evaluate the impact scenarios to climate phenomena.

In^15^ the combination of exposure and the vulnerabilities of each subsystem (called Intrinsic Vulnerabilities) is measured with a specific indicator labelled Combined Vulnerability. The impact indicator is measured as a merger of the Combined Vulnerability and the hazard scenario (Fig. 1). The exposure is valuated in two phases: in the former phase (phase 1) the elements exposed to the climate risk are defined, and in the latter phase (phase 2) the exposure of those elements is measured.

The partitioning of the urban pattern into subsystems is done to separate and recognize the elements and components that make it up. Since each subsystem is taken into consideration regardless of its relationship with the other urban subsystems, this partitioning simplifies the assessment of the vulnerability of the urban system. The vulnerability of each subsystem is measured by considering only the characteristics of the subsystem affected by the heatwave phenomenon.

This model is applied in^15^ to evaluate the impact heatwave scenarios. The urban system is partitioned into three subsystems: residential buildings, open spaces, and population; the model was implemented and tested to evaluate the vulnerability of the three subsystems to the heatwave phenomenon and the impact scenarios. The model was evaluated in accordance with the research reported in^17,18^, considering the resident population as an exposure component and assessing the combined vulnerability by combining the intrinsic vulnerabilities of the subsystems with the exposure.

The characteristics of the subsystem elements that might affect a subsystem's response to heatwave events are assessed in order to evaluate the intrinsic vulnerability of the subsystems. They refer to the construction, morphological, technological, and environmental aspects of residential buildings and open spaces (eras and construction techniques, volume and solar exposure of the building envelope for the residential buildings, and, Sky View Factor, solar exposure, Albedo and NDVI for the open space). In^19^ are applied recent remote sensing techniques aimed at the acquisition and synthesis of the NDVI. In similar ways, the same techniques are used to calculate the characteristics of the physical system that have as a source given satellite images (Albedo, Sky View Factor, etc.).

The assessment of heatwave impact scenarios takes account of the major risk factors for residents who are most inclined to suffer heat exhaustion and other health effects because of the phenomenon. The exposure is measured by computing the number of residents living in each residential building. The hazard scenarios are computed from the spatial distribution of the raster satellite data of the surface temperature at both daily and nightly times measured during heatwave periods. The impact scenarios are calculated as a combination of a hazard scenario and a combined vulnerability.

### Process employed to assess heatwave vulnerability and impact scenarios

This section briefly presents the model proposed in^15^, chosen as the starting point for the elaboration of our framework. The overall process is detailed in^15^ and it was implemented on a GIS platform.

**Figure 2:**
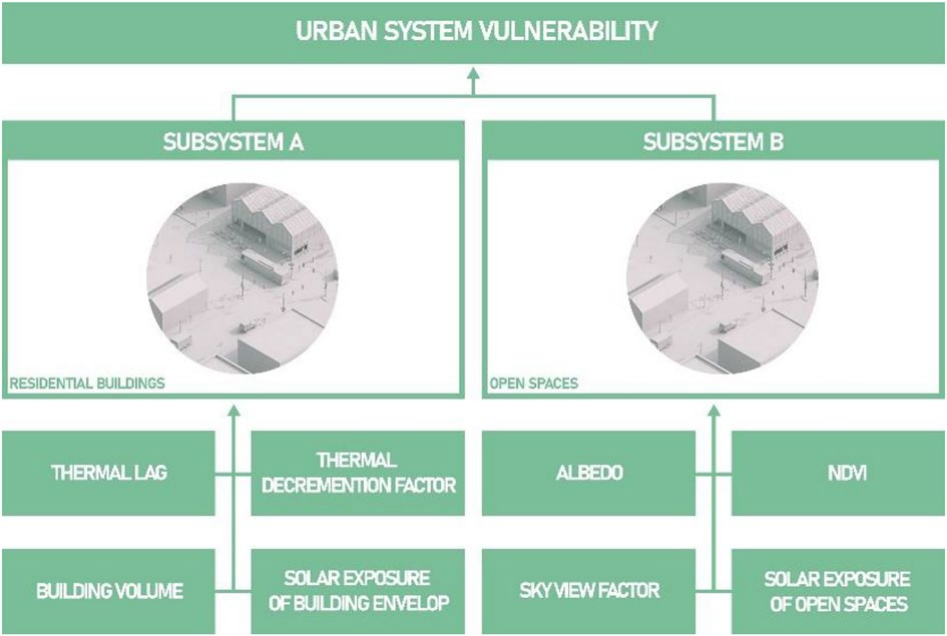
Schema of the indicators used in^15^ to evaluate the residential buildings and open spaces heatwave vulnerability.

To evaluate the vulnerability of the residential building and open space subsystems, a set of indicators was computed that recognizes the main characteristics of the settlements in the type-morphological and technological aspects, as well as in the presence and intensity of greenery and urban elements capable of affecting the aspects of temperature, ventilation, and relative humidity during intense climatic phenomena^20^. These indicators are shown in Fig. 2.

Each indicator is obtained by a measure of a characteristic of buildings and open spaces; to each indicator is assigned a weight based on how much it affects the intrinsic vulnerability of the subsystem. The weights are assigned in^15^ following a specific calibration (Table 1).

**Table 1:** Indicators of physical subsystem.

Subsystem	Indicator	Measure	Measure domain	Weight
Buildings	Thermal lag	Thermal lag (s)		5
	Thermal decrement factor	Thermal decrement factor	[0, 1]	4
	Building volume	Building volume (m^3^)		2
	Solar exposure of building envelope	Façades and roofs Hillshade	[0, 255]	5
Open spaces	SVF	Sky View Factor	[0, 2π]	5
	Albedo	Albedo	[0, 1]	2
	Solar exposure of open spaces	Open space Hillshade	[0, 255]	4
	NDVI	Normalized Difference Vegetation Index	[− 1, 1]	3

The indicators are partitioned into five classes from 1 to 5, making them comparable to each other to normalize the impact assessment operations. All indicators foresee a partition into five classes (Table 2). The partition rules are obtained through calibrations on specific samples^15^.

**Table 2:** Classification of indicators.

Class	Label
1	High
2	Medium–high
3	Medium
4	Medium–low
5	Low

The combined vulnerability is computed by merging the contribution of the exposure, given by the resident population living in a residential building, the intrinsic vulnerability of the residential buildings, and the mean intrinsic vulnerability of the surrounding open spaces.

The hazard features that constitute the hazard scenario are evaluated and distributed over the study region in a collection of raster data. The spatial model used to realize the scenarios was designed to be inter-scalable and portable. In fact, regardless of the spatial accuracy and amount of input data available in the research region, it may be used on any sort of geographical scale. The hazard scenarios are constructed by considering the projected variations in maximum and minimum temperatures, Heat Index and surface temperature, according to two IPCC emission scenarios:

- RCP 4.5 scenario which provides for a constant concentration of greenhouse gases until 2100;
- the RCP 8.5 scenario which provides for a constant increase in concentration of greenhouse gases up to 2100.

The IPCC RCP 4.5 model predicts that greenhouse gas emissions will slow down over the next 50 years but that their concentrations in the atmosphere will continue to increase. The choice of this model is related to its degree of likelihood, as the current trend suggests a decrease in greenhouse gas emissions from human activities, so that the radiative forcing may reach the value of 4.5 W/m^2^ with a consequent increase in average temperature of less than 2 °C. The RCP4.5 model assumes an almost linear trend with a consequent constant temperature growth until reaching 2 °C in 50 years. From the study carried out by Euro-Mediterranean Center on Climate Change (CMCC) on the city of Naples (https://www.cmcc.it/it/report-napoli), the projections of the two models have a similar trend until 2050 and then vary more and more until 2100, with a final average change of about 2 °C. The Root Mean Square Error (RMSE) value obtained by comparing the temperature difference between the models' projection and the actual measurement recorded over the past 5 years is below 0.1 °C. Furthermore, the RCP4.5 model has a standard deviation from the projected average summer temperature up to 2050 of about 1 °C and then increases to 1.5 °C by 2100.

The hazard is analyzed based on three different scenarios for each of the two RCP models: a short-term, a medium-term, and a long-term scenario. The impact of the heatwave phenomenon is assessed for each hazard scenario by combining the hazard and Combined Vulnerability (on a scale from one, more serious, to five, less severe).

The impact scenarios were evaluated by combing on one side with the Combined Vulnerability, which is used as a fixed, non-mutable part that represents the conditions that lead to the urban environment, and on the other side with all the hazard scenarios, which possess a greater dynamism as they represent the evolution of the danger during a given period.

## Materials and methods

### The study areas

Our framework was tested on the study area of the dense city of Naples (Italy) and subsequently, in order to verify its portability in other urban fabrics, it was tested on the city of Avellino (Italy), a different urban fabric less populated and characterized by different climatic conditions than Naples.

The municipality of Naples is populated by a little less than a million inhabitants, it represents a complex urban fabric with a high population density. It possesses a multiplicity of different urban forms, which connote a certain level of homogeneity. In fact, it is possible to identify cohesive areas with high construction density, such as those present in the historic center, areas with a lower concentration of buildings and a clear prevalence of forested areas, such as the peripheral areas to the north, areas with greater extension and a prevalence of manufacturing building artefacts, such as the areas to the east with a strongly industrial vocation.

A study about climate change carried out in^21^ predicts that all of Italy's coastal areas will be marked by a temperature increase in the period 2021–2050, compared to 1981–2010, and that this increase equates to 1.3 °C in the Central and Western Mediterranean.

Naples is among the Italian cities with the highest percentage of artificial surface compared to administrative thresholds, at around 63%^22^; in addition, the city of Naples, comprising about one million inhabitants, is classified as the Italian urban area with the highest density of inhabitants per square kilometer. These factors make the city of Naples particularly vulnerable to heatwave impact scenarios.

The maximum temperature anomalies observed in the city in 2018 were about 0.9 °C lower than those recorded in the period 1971–2000^23^. In contrast, despite the drop in maximum temperature anomalies, the number of tropical nights has grown dramatically, with an increase of about 37 days over the course of the current season compared to the median of the reference period^24^.

In order to test the performance of our framework on different urban fabrics, we replicated the processes used to create the resident building vulnerability in the city of Avellino.

Avellino, in contrast to Naples, is characterized by a less complex urban fabric, a substantially smaller overall population (about 52,000), and a population density that is five times lower. Less compact buildings provide the benefit of having distributed open areas. The core of the building itself is surrounded by a predominantly natural territory with a strong mountainous character. This directly affects the climate, which offers lower temperatures than the city of Naples but nevertheless remains subject to extreme weather events such as heatwaves (on average, in the summer months, the max air temperature is 28.7 °C; in July and August, it is about 30 °C).

### Methodological framework

Our framework was developed starting from the model^15^ and it was integrated with an approach aimed at achieving the reusability capabilities of the model in order to be applied in different urban fabrics.

In order to better analyze vulnerabilities and impacts, it was chosen to employ a smaller spatial scale than that specified in the model of^15^, which set up building and open space polygonal entities as atomic units. We have chosen to use the census section as an atomic entity since it represents the smallest area recognized as homogenous regarding urban characteristics and is frequently updated by the national census bodies. This choice was made to guarantee the usability of our framework even in urban fabrics where the data at this scale are not accessible and to provide a trade-off between the assessment accuracy and the portability of the model.

**Figure 3:**
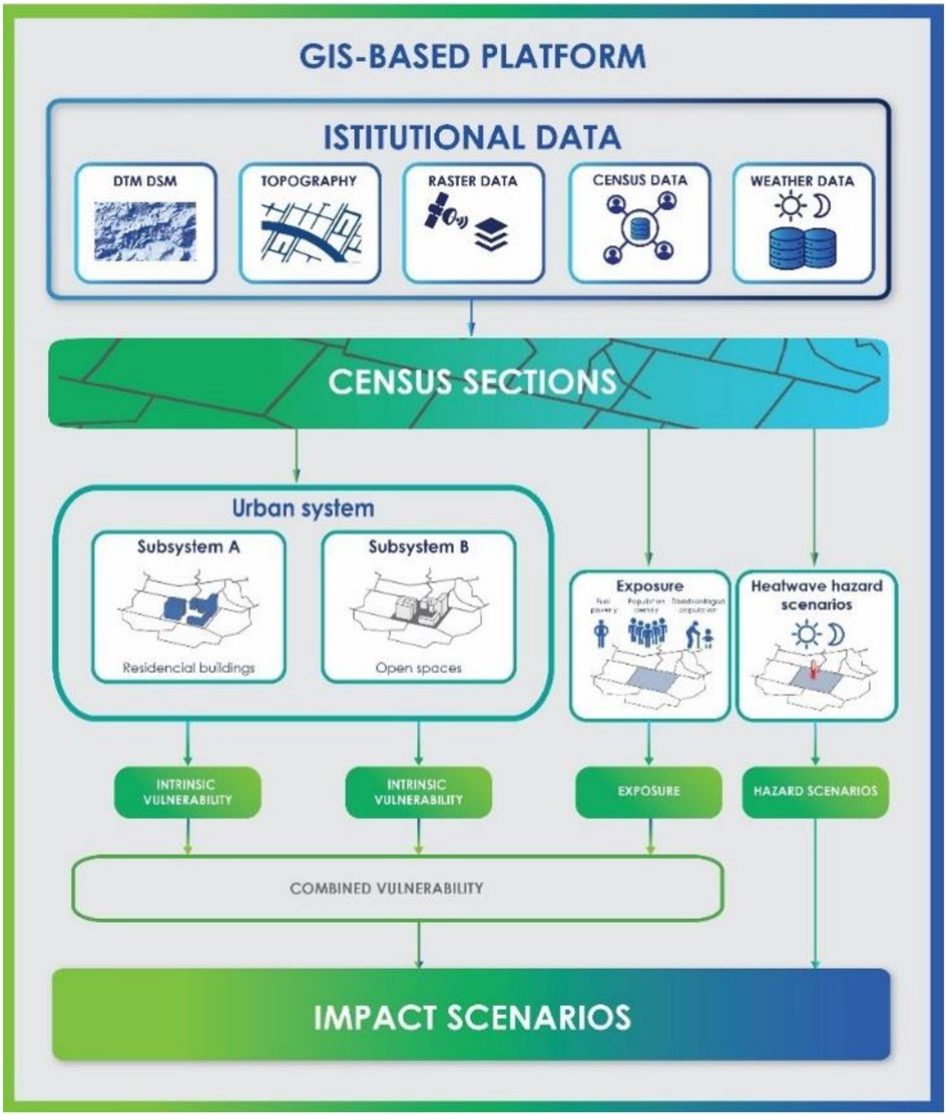
Model for assessing impact scenarios for climatic phenomena.

Figure 3 shows the entire structure of the framework and the connections between its parts to determine the heatwave impact scenarios.

The intrinsic vulnerability of the urban physical subsystems, the exposure, and the hazard scenarios generated by heatwave phenomena are computed using input data from certified sources, where for intrinsic vulnerability we mean the vulnerability of each subsystem. The combined vulnerability is then calculated by combining the Exposure with the building and open spaces intrinsic vulnerabilities. Finally, an Impact scenario is created by merging the effects of a hazard scenario with the combined vulnerability.

The buildings and open spaces Intrinsic Vulnerabilities are computed by using the indicators in Fig. 2 and applying the processes described and synthetized in "Preliminaries" section.

To calculate exposure, we focus on the population density and identify categories of residents who are most vulnerable to the risks linked to the heatwave phenomena. In fact, the population is subject to discomfort and possible damage to health caused by the occurrence of the phenomenon^25–27^. The exposure is computed starting from the population census data for census sections provided and updated by a national statistical institute (e.g., the Italian statistical institute of ISTAT in Italy, the Office for National Statistics in UK, the French national institute of statistics and economic studies INSEE in France, the Spanish statistical office INE in Spain, etc.). It was allowed to individually assess the impact scenarios produced by different categories of inhabitants exposed to risk since exposure, in the model created for calculating the impacts, is an independent variable from the other entities of the model. We have considered three types of exposure:

- Population density (PD); aimed at identifying the spread of the population in the study area and highlighting the degree of crowding in that area;
- Disadvantaged population (DP); aimed at identifying the spread in the territory of the population belonging to the weaker groups in relation to age (children and the elderly) and the relative crowding in certain sections of the census;
- Fuel Poverty (FP); aimed at identifying the spread over the territory of the population in particularly conditions disadvantaged people (households > 5 members, non-income earners, unemployed) and the relative crowding in certain sections of the census.

The PD is expressed in inhabitants per square kilometer, and it is determined using the following formula:

1 $$PD= \frac{P}{S{\text{S}}}$$

where P is the number of residents in the census section and SS is the area of census section in km^2^.

The indicator is obtained by using the partitioning of the PD index in five classes, following the one used in^15^. That partitioning is shown in Table 3

**Table 3:** Classification of the population density indicator.

Rule	Class	Label
PD ≥ 20,000	1	High
15,000 PD < 20,000	2	Medium–high
10,000 ≤ PD < 15,000	3	Medium
5000 ≤ PD < 10,000	4	Medium–low
PD < 5000	5	Low

The DP is expressed in inhabitants per square kilometer and it is computed using the following formula:

2 $$DP= \frac{{P}_{D}}{S{\text{S}}}$$

where *P*_*D*_ is the number of residents in the census section with younger than 6 years and older than 65 years, and *S*_*S*_ is the area of census section.

The disadvantaged population indicator is obtained by using the partitioning of the DP index in five classes, following the one used in in^15^. That partitioning is shown in Table 4

**Table 4:** Classification of the disadvantaged population indicator.

Data representation	Class	Label	Classification method
DP ≥ 5000	1	High	Manual
5300 ≤ DP < 5000	2	Medium–high	Manual
2000 ≤ DP < 5300	3	Medium	Manual
500 ≤ DP < 2000	4	Medium–low	Manual
DP < 500	5	Low	Manual

The FP is expressed in inhabitants per square kilometer, and it is computesd using the following formula:

3 $$FP= \frac{{N}_{F}+N + D}{S{\text{S}}}$$

where *N*_*F*_ is the number of households whit more than five members, N is the number of non-recipients of income, D is the number of unemployed and *S*_*S*_ is the area of census section.

The fuel poverty indicator is obtained by using the partitioning of the FP index in five classes, following the one used in^15^. That partitioning is shown in Table 5

**Table 5:** Classification of the fuel poverty indicator.

Data representation	Class	Label	Classification method
FP ≥ 5000	1	High	Manual
5300 ≤ FP < 5000	2	Medium–high	Manual
2000 ≤ FP < 3500	3	Medium	Manual
500 ≤ FP < 2000	4	Medium–low	Manual
FP < 500	5	Low	Manual

Since it has been considered that residents interact primarily with the material components of the urban system, residential buildings and open spaces constitute all the subsystems with which exposure interacts^13^. In our framework, in which the atomic entity is the census section, the values ​​of the two physical subsystems indicators are aggregated considering all the components of the subsystem located in the census section.

The combined vulnerability merges the exposure with the intrinsic vulnerability of the subsystems. A set of calibration processes were performed in^15^ to extract the combined vulnerability as a function of the exposure and intrinsic vulnerabilities of the two physical subsystems. Table 6 shows the calibration used to obtain the combined vulnerability values.

**Table 6:** Combined vulnerability indicator.

Residential buildings intrinsic vulnerability	Open spaces intrinsic vulnerability	Exposure	Combined vulnerability
< 3	< 3	< 3	1
< 3	≥ 3	< 3	2
< 3	< 3	≥ 3	2
< 3	≥ 3	≥ 3	3
≥ 3	< 3	< 3	3
> 3	≥ 3	< 3	3
= 3	≥ 3	< 3	3
= 3	< 3	≥ 3	3
> 3	< 3	≥ 3	4
= 3	≥ 3	≥ 3	4
> 3	≥ 3	≥ 3	5

The features that connote a hazard scenario can be divided into spatial and time features; the time features are given by the following daily climatic parameters: the maximum and minimum air temperatures and the relative humidity. These three parameters are used to compute the Heat Index (HI) measure defined by the USA National Weather Service. A heatwave scenario is detected by the presence of a HI higher than 32 °C for at least three consecutive days^28^.

The hazard spatial features are given by the remote sensed surface temperature raster data collected during a heatwave and monitored both daily and at night. The most dangerous areas are detected measuring the difference between the day and overnight temperatures. The hazard increases as this difference decreases.

The assessment of the hazard is obtained by combining time and spatial features, following the processes described in^15,17,18^ in which are considered three types of hazard scenarios: short-term (2020–2040), medium-term (2041–2070), and long-term (2071–2100).

To each scenario is assigned a mean number of heatwave consecutive days: short-term 6 days, medium-term 30 days, and long-term 60 days.

In a short-term scenario, in which the duration of heatwave consecutive days ranges between 3 and 8 days, the hazard indicator obtained using Table 7^15,18^:

**Table 7:** Hazard classification in a heatwave short-term scenario.

Surface temperature difference	Hazard class	Label
ΔT ≤ 7	1	High
7 < ΔT ≤ 10	2	Medium–high
10 < ΔT ≤ 15	3	Medium
15 < ΔT ≤ 20	4	Medium–low
ΔT > 20	5	Low

where ΔT is the surface temperature difference between day and night.

The variations of the impact classes are evaluated with the worsening of the phenomenon, using the same calibration criteria proposed in^15^. Table 8 shows the respective variations for each hazard class as the scenario worsens. For example, an urban area classified with a Medium–low hazard (4), in a short-term scenario, will be assigned to the hazard Medium (3) class in a medium-term scenario, and to High class (1) in a long-term scenario. This implies that areas with Low hazard values, determined in a short-term scenario without appropriate mitigation and/or adaptation interventions, are transformed into High hazard areas in a long-term scenario.

**Table 8:** Change of the hazard classes on varying the hazard scenario.

Short-term		Medium-term		Long-term	
Class	Label	Class	Label	Class	Label
1	High	1	High	1	High
2	Medium–high	1	High	1	High
3	Medium	2	Medium–high	1	High
4	Medium–low	3	Medium	1	High
5	Low	4	Medium–low	2	Medium–high

This assessment considers adaptive and mitigation changes to the urban setting do not occur until 2100.

**Figure 4:**
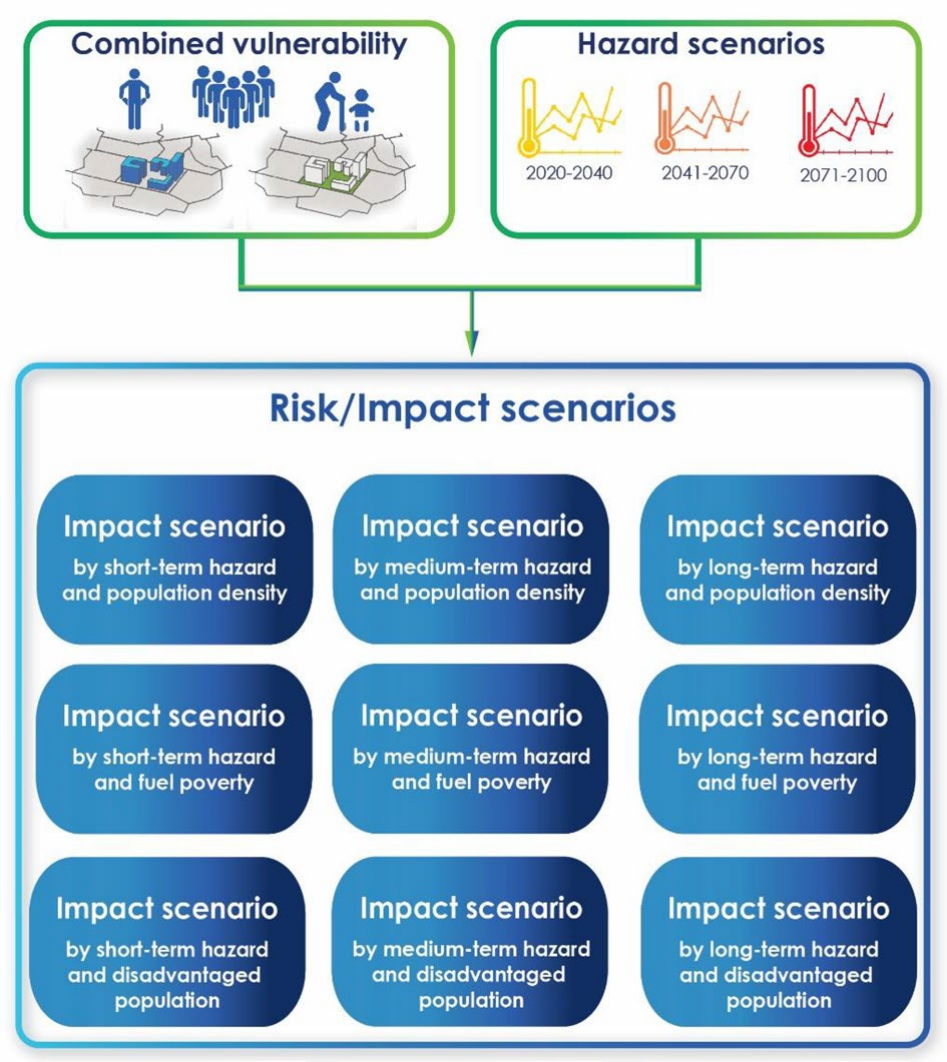
Risk/Impact scenarios evaluating by different exposition.

Impact scenarios are computed by combining hazard scenarios with exposure. A different impact scenario is generated depending on the hazard discharge and the type of population exposed. Combining the two indicators of hazard and combined vulnerability has resulted in the creation of three impact scenarios—one for each of the obtained thematic maps of integrated vulnerability—for each hazard scenario. Overall, nine impact scenario maps are produced: three short-term, three medium-term and three long-term (Fig. 4).

To evaluate the functional dependence of the impact class on both hazard and vulnerability indicators, a specific calibrations rule set proposed in^15^ is used. It is schematized in Table 9.

**Table 9:** Impact class according with combined vulnerability and hazard classes.

Hazard class	Combined vulnerability class	Impact class
< 3	< 3	1
≥ 3	< 3	2
< 3	≥ 3	Combined vulnerability class—1
≥ 2	≥ 3	Combined vulnerability class

Following this calibration rule set, if both hazard and combined vulnerability are High or Medium–high the impact class is set to High. Otherwise, if the combined vulnerability is at least Medium (Medium, Medium–low or Low), and if also the hazard is at least Medium–high, the impact class is identical to the combined vulnerability class; finally, it is given by the combined vulnerability class values  − 1.

## Results and discussion

### Results

The model has been structured and implemented in a GIS platform using the suite GIS ESRI ArcGIS Desktop. The spatial analysis processes were implemented using the ArcGIS tool ModelBuilder, a visual programming language for building geoprocessing workflows that generate geoprocessing models that automate and document spatial analysis and data management processes.

#### Vulnerabilities and impact scenarios on the municipality of Naples (Italy)

To execute our framework the following datasets have been imported:

- Digital Terrain Model (DTM) and Digital Surface Model (DSM) obtained from LIDAR surveys provided by the Italian Ministry and provided by the Italian Ministry of the Environment and Protection of Land and Sea in raster format, with a resolution of 1 m × 1 m;
- Topographic database of the study areas, processed and released in shapefile format by the Campania Region with a geographic scale of 1:500;
- National Census Data in shapefile format with a scale of 1:10,000 processed and supplied by the National Institute of Statistics (ISTA) and constantly updated;
- Raster Albedo processed using the remote sensed Sentinel2 multi-band images, with a resolution of 7 m × 9 m;
- Raster Sky View Factor processed using the remote sensed Sentinel2 multi-band images, with a resolution of 1 m × 1 m;
- Raster NDVI, processed using the remote sensed Sentinel2 red and near infrared bands, with a resolution of 7 m × 7 m;
- Satellite data of daytime and night-time Land Surface Temperature (LST) processed using the remote sensed Landsat8 multi-band images, with a resolution of 30 m × 30 m.

The satellite raster data are necessary to extract the spatial distribution of some parameters, as Albedo, NDVI, and surface temperatures, to compute intermediate indicators; all are single images and were processed during the occurrence of the heatwave phenomenon in Naples on 14/07/2020.

**Figure 5:**
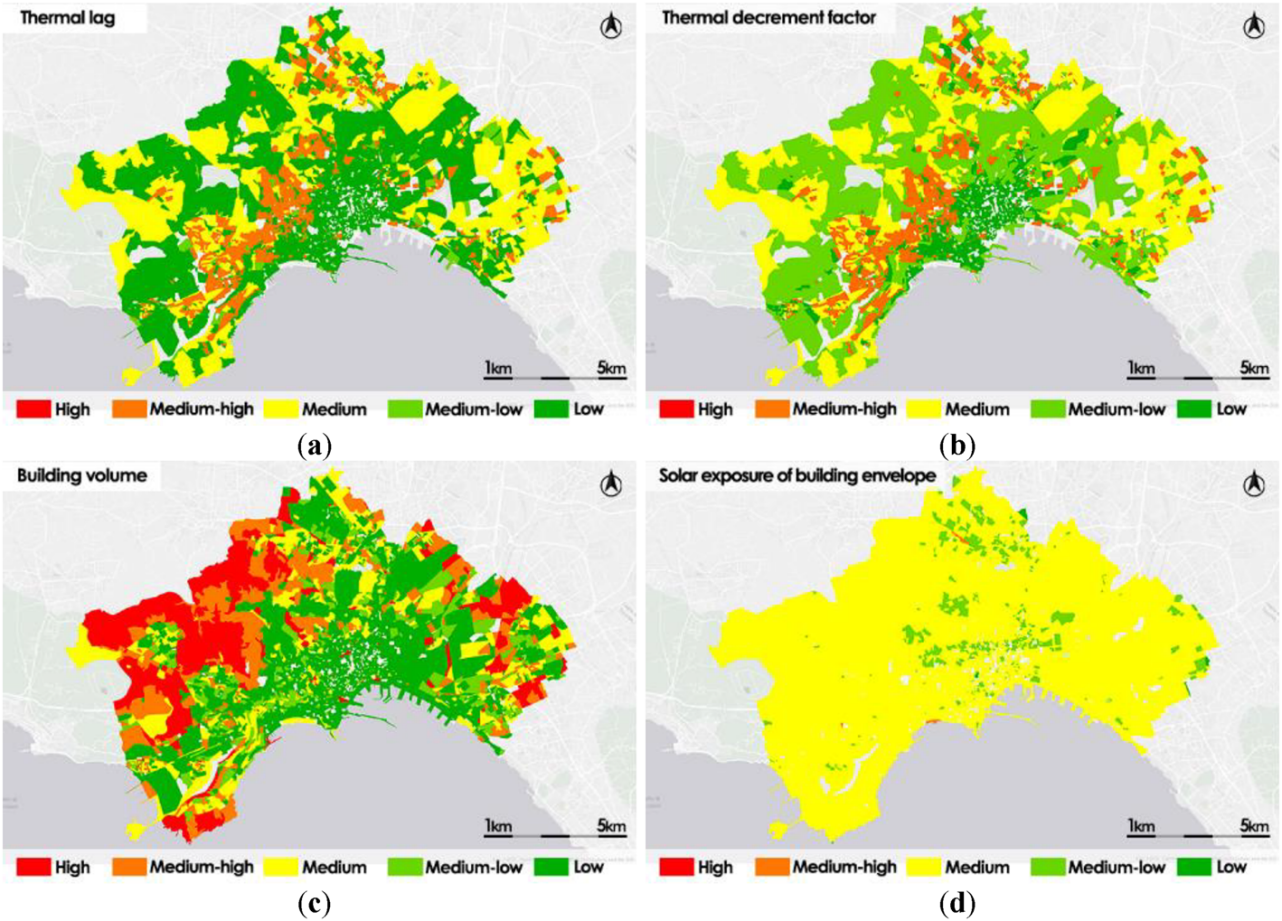
Residential building subsystem vulnerability intermediate indicators: Thermal lag (**a**); Thermal decrement factor (**b**); Building volume (**c**); Solar exposure of building envelope (**d**).

Figure 5 shows the thematic maps of the residential building subsystem indicators: Thermal lag, Thermal decrement factor, Building volume and Solar exposure of building envelope.

The Thermal lag map shows the presence of census sections with Medium–high value in some areas to the north and mainly in the districts of the central-western areas of the municipality of Naples. Similarly, in these zones the thematic map of the Thermal decrement factor shows a Medium–high criticality. The Building volume indicator thematic map shows that the census areas containing the most voluminous residential buildings are mainly located in the peripheral areas of the city, especially in the north-western area. The Solar exposure of building envelope thematic map presents a uniform distribution throughout the municipal area with over 90% of the sections classified as Medium.

**Figure 6:**
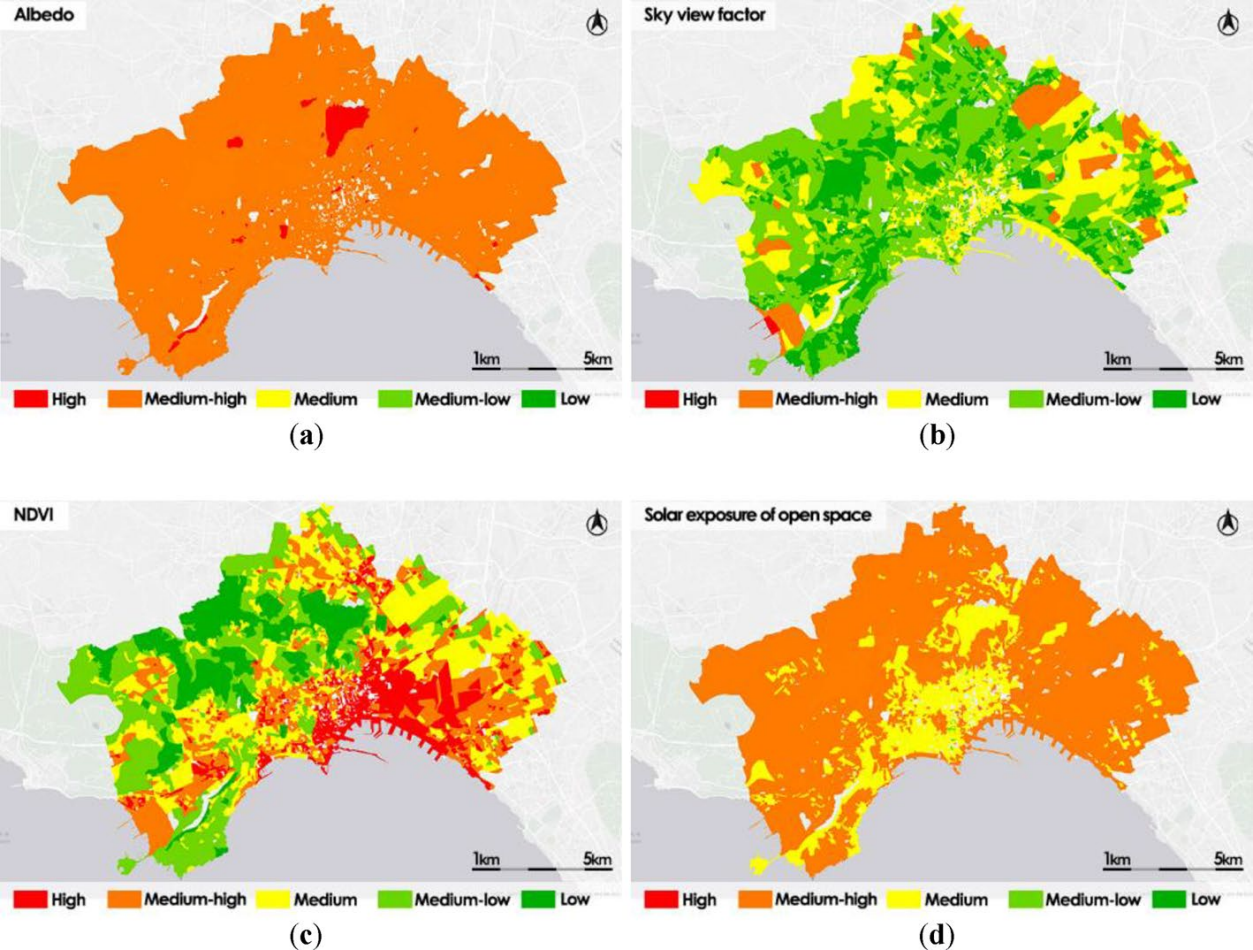
Open spaces subsystem vulnerability indicators: Albedo (**a**); Sky view Factor (**b**); NDVI (c); Solar exposure of open space (**d**).

The thematic maps shown in Fig. 6 are the outcome of the process required for computing the intermediate vulnerability indicators of the open space subsystem.

The Albedo thematic map shows a distribution almost uniform in which most of the census sections are classified as Medium–high or High, with a distribution of greater criticality located in the historic center. In the Sky view factor thematic map, most of the census sections are classified as Low or Medium–low; this is mainly due to the prevalence of census sections in which the portion of the visible sky in the open space is reduced by the presence of neighboring buildings and other vertical structures. In the NDVI map, most of the densely inhabited census sections and the historic center, which are poor in living vegetation, are classified as High or Medium–high; conversely, the north-western census areas, which enjoy the greatest presence of plant species, are classified as Medium–low or Low. In the Solar exposure thematic map, the census sections located in the city center are prevalently classified as Medium, in contrast to the census sections of the peripheral areas, which are classified as Medium–high.

**Figure 7:**
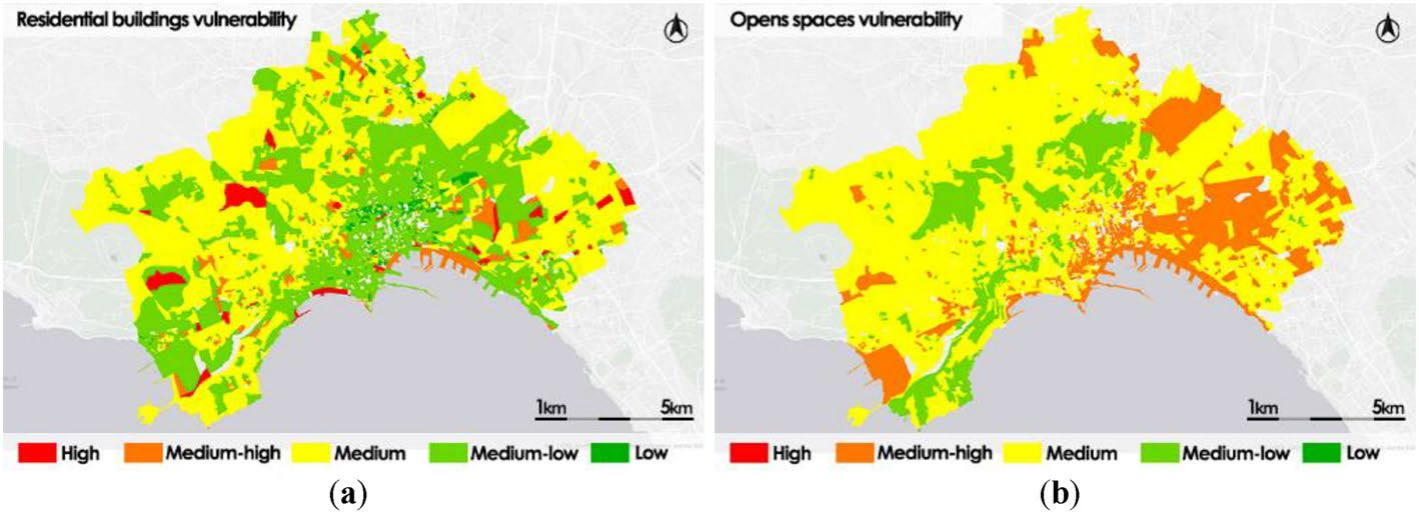
Urban system intrinsic vulnerabilities: residential building vulnerability (**a**); open spaces vulnerability (**b**).

The thematic maps presented in Fig. 7 are the outcome of the process for calculating the intrinsic vulnerabilities of the urban system.

The residential building vulnerability thematic map shows a prevailing condition of Medium or Medium–low vulnerability throughout the area of the municipality of Naples (91% of the census sections). In particular, in the historic center there is an extended area covered by census sections with Low or Medium–low vulnerability.

In the open spaces subsystem vulnerability map the east side of the city is prevalently covered by census sections classified as Medium–high. In the western and central-western areas, census sections classified as Medium vulnerability class prevail, whereas only a few census sections, mainly covered by vegetated areas, are classified with Medium–low vulnerability.

**Figure 8:**
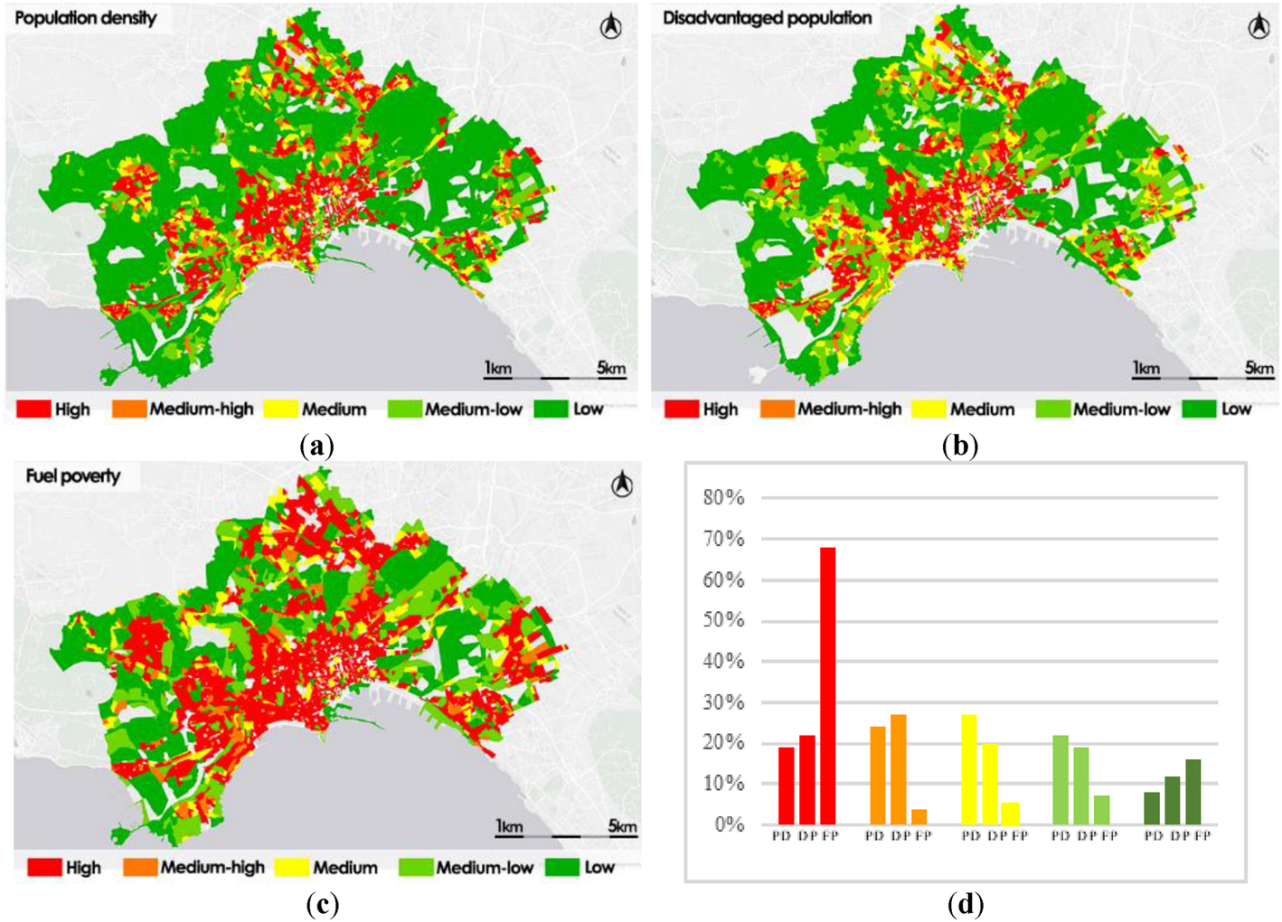
Exposure to heatwave phenomena: Population density (PD) (**a**); Disadvantaged population (DP) (**b**); Fuel poverty (FP) (**c**); frequency histogram (**d**).

In Fig. 8 are shown the thematic maps obtained for the three types of exposure analyzed correspond to the three categories of population: Population density, Fuel poverty and Disadvantaged population.

The population density map shows that the areas of the historic center and some suburban districts are more exposed, justified by a greater population density. About 43% of the census sections have High or Medium–high exposure, while only 30% of them is classified as Medium–low or Low.

The spatial distribution of the disadvantaged population exposure is similar to Population density distribution; the areas most affected are mainly located in the historic center of the city and some peripheral districts; about 49% of the census sections are classified as High or Medium–high.

The fuel poverty thematic map presents more critical issues than the two other exposure thematic maps. In fact, it shows larger areas with high exposure value in the historic center and in some peripheral areas: 72% of the census sections are classified as High or Medium–high. Only 23% of the census sections are classified as Low or Medium–low.

To develop the impact scenarios is considered the IPCC RCP 4.5 model which predicts that greenhouse gas emissions would slow down over the next 50 years but that their atmospheric concentrations will continue to rise. the reason for choosing this model is that the RCP 4.5 model is the one that seems more probable today as the current trend suggests a decrease in greenhouse gas emissions produced by human activities, so that the radioactive forcing will not be able to reach the value of 8.5 W m^2^ as in the RCP 8.5 model, even if it will not be such as to avoid global warming of the planet with an average temperature increase of less than 2 °C. For this reason, we decided to focus the heatwave impact scenarios on relation to hazard scenarios, taking into account the RCP 4.5 model, in which the average duration of a heatwave is 6 days in the short-term, 30 days in the medium-term and 60 days in the long-term.

**Figure 9:**
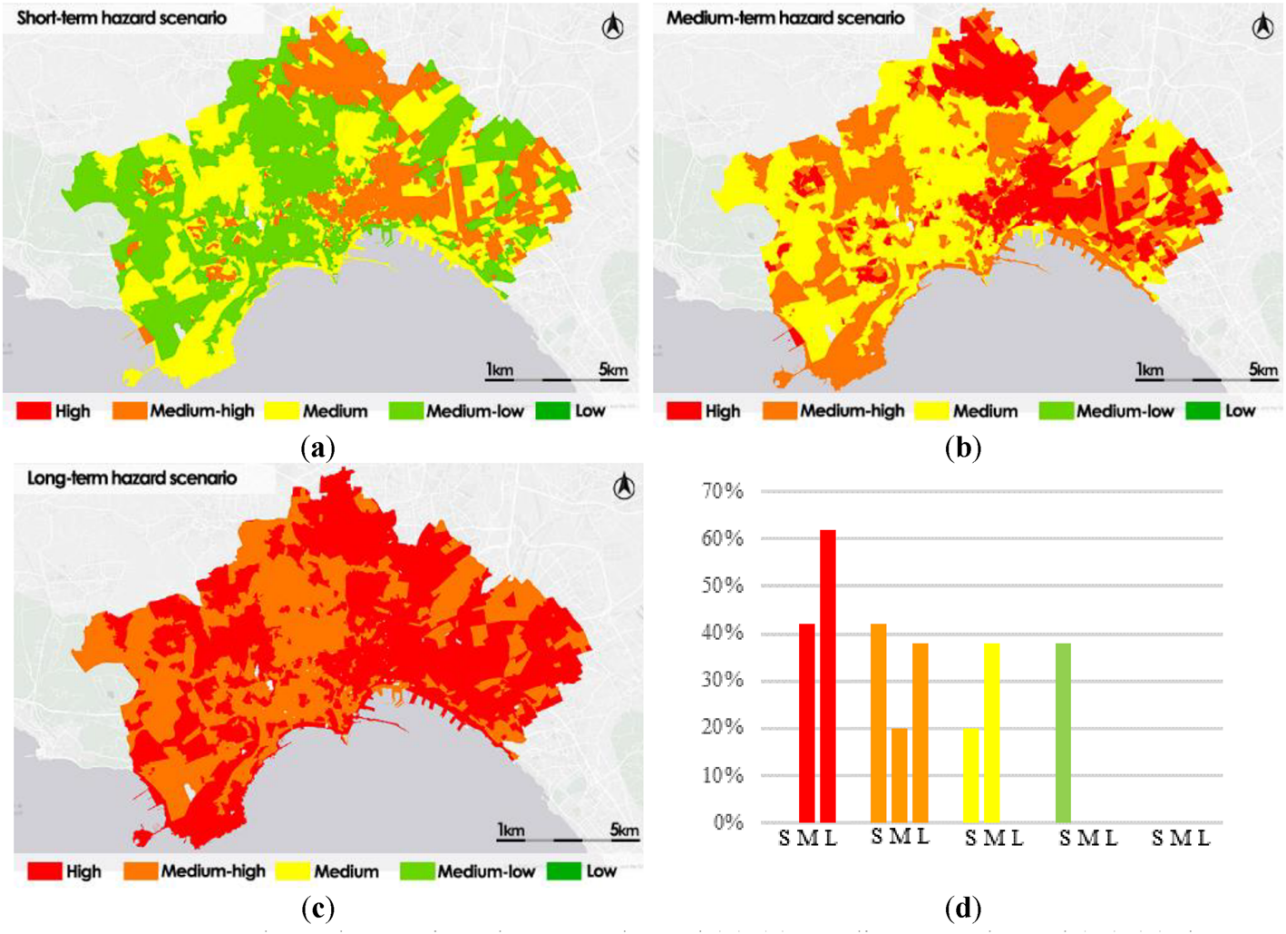
Heatwave hazard scenarios: short-term hazard (S) (**a**); medium-term hazard (M) (**b**); long-term hazard (L) (**c**); frequency histogram (**d**).

Figure 9 shows the thematic maps corresponding to the three-time scenarios: short-term, medium-term, and long-term.

As seen in the study of the three hazard scenario maps, the threat of the phenomenon increases over time, becoming highly significant in the long run with High and Medium–high hazard over the study area.

The histogram in Fig. 9d shows the evolution of the hazard in the three scenarios. Starting from the short-term scenario, about 58% of the census sections are classified as Medium–low or Medium. In the medium-term scenario the distribution worsens by one class and the percentage of census sections classified as Medium is reduced to 38%; conversely, the percentage of High and Medium–high which increases to 62%. In the long-term scenario, there are no longer sections belonging to the Medium or higher classes, 100% of the sections reach High or Medium–high hazard levels. This conclusion shows that, unless adaptation measures are planned, the phenomena will reach a very critical level by 2100, affecting the entire study area, independent of the diverse typologies of the portions that make it up.

**Figure 10:**
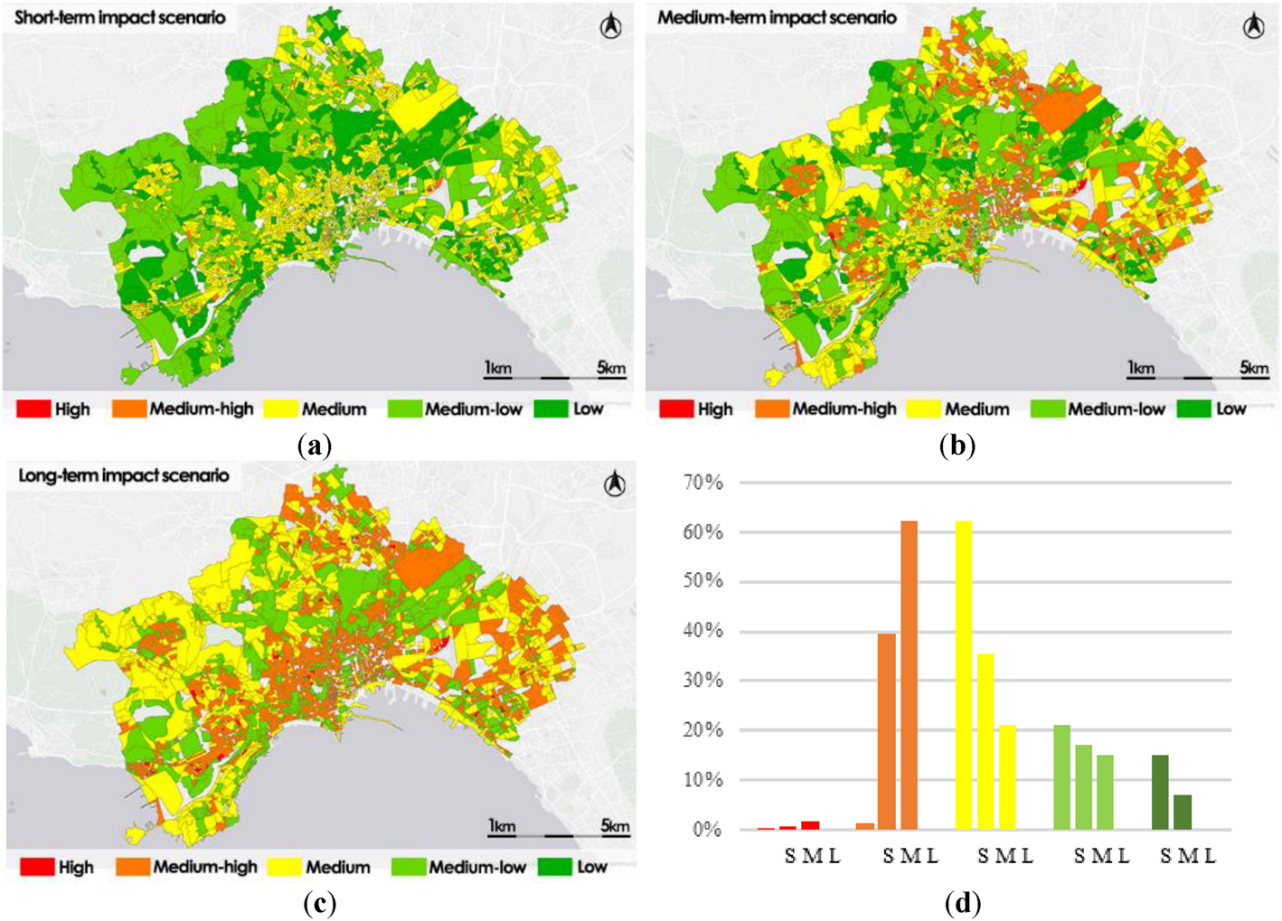
Impact scenarios by population density: short-term impact scenario (S) (**a**); medium-term impact scenario (M) (**b**); long-term impact scenario (L) (**c**); frequency histogram (**d**).

The thematic maps of the three impact scenarios have been drawn up for each hazard scenario by each category of exposure. In Fig. 10 it is illustrated the impacts maps by Population density.

The short-term impact scenario map shows a higher percentage of census sections belonging to Medium class (39%), the census sections that have the most crucial difficulties is also the 33% classified as High or Medium–high, only the 28% of sections has a good level of impact, classified as Medium–low or Low.

In the medium-term scenario map the distribution is different, there is a worsening of the condition of the impact levels with the census sections percentage belonging to the highest levels classified as High or Medium–High reachings 43%, while the census sections percentage classified as Medium decreases to 34% and that classified as Medium–low or Low decreases to 23%.

In the long-term scenario maps the impact levels worsen dramatically, compared to the initial conditions, the census sections classified as High or Medium–high reach 66%, sections percentage classified as Medium decreasse to 20% and that classified as Medium–low is reduced to 14%, there is no census section belonging to the Low level.

The most critical areas are mainly distributed in the historic center and in the northern and eastern surrounding areas. The western zones in the longer scenario maintain conditions of Medium or Medium–low levels.

**Figure 11:**
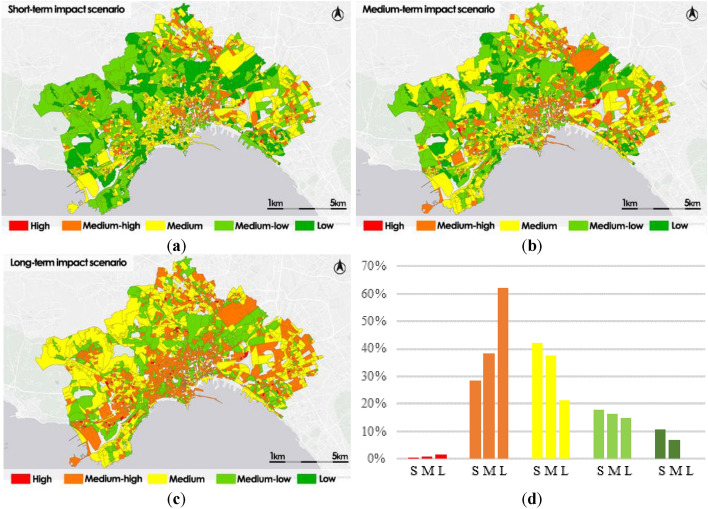
Impact scenarios by disadvantaged population: short-term impact scenario (S) (**a**); medium-term impact scenario (M) (**b**); long-term impact scenario (L) (**c**); frequency histogram (**d**).

In Fig. 11, it is possible to see the impacts maps for each hazard scenario by disadvantaged population.

The short-term impact scenario map shows a higher percentage of census sections belonging to Medium class (48%), the census sections that have the most crucial difficulties is also the 38% classified as High or Medium–high, only the 14% of sections has a good level of impact, classified as Medium–low or Low.

In the medium-term scenario map the distribution is different, there is a worsening of the condition of the impact levels with the census sections belonging to the highest levels classified as High or Medium–high reaching 52%, while the census sections classified as Medium decrease to 36% and those classified as Medium–low or Low decrease to 12%.

In the long-term scenario maps the impact levels worsen dramatically, compared to the initial conditions, the census sections classified as High or Medium–high reach 84%, the sections percentage classified as Medium decreases to 9% and that classified as Medium–low is reduced to 7%, there is no census section belonging to the Low level.

The most critical areas are mainly distributed in the historic center and in the northern and eastern peripheral areas. The north–western zones in the longer scenario maintain conditions of Medium or Medium–low levels.

**Figure 12:**
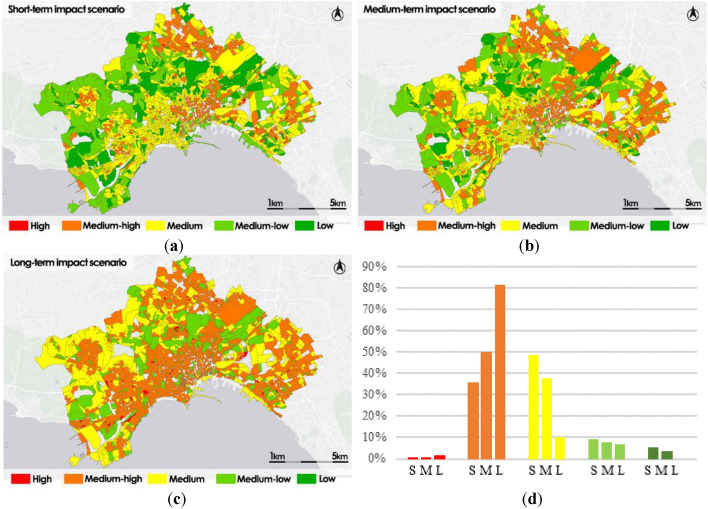
Impact scenarios by fuel poverty: short-term impact scenario (S) (**a**); medium-term impact scenario (M) (**b**); long-term impact scenario (L) (**c**); frequency histogram (**d**).

Figure 12 shows the distribution of impact classes in each hazard scenario by the fuel poverty population.

The short-term impact scenario map shows a higher percentage of census sections belonging to Medium class (41%), the census sections that have the most crucial difficulties is also the 31% classified as High or Medium–high; only the 28% of them all has a good level of impact, classified as Medium–low or Low.

In the medium-term scenario map the distribution is different, there is a worsening of the condition of the impact levels with the census sections belonging to the highest levels classified as High or Medium–High reaching 41%, while the census sections classified as Medium decrease to 36% and those classified as Medium–low or Low decrease to 23%.

In the long-term scenario maps the impact levels worsen dramatically, compared to the initial conditions, the census sections classified as High or Medium–high reach 65%, those classified as Medium decrease to 21%, and those classified as Medium–low are reduced to 14%; there is no census section belonging to the Low level.

The most critical areas are mainly distributed in the historic center and in the northern and eastern peripheral areas. The north–western zones in the longer scenario maintain conditions of Medium or Medium–low levels.

By examining the various impact maps, it is feasible to see that in the medium-term and long-term scenarios, over 40% of the census sections already experience significant criticalities. These percentages reach 52% in the medium-term scenario and 84% in the long-term scenario, specifically in the impact scenarios by people living in fuel poverty.

The results also reveals that the fuel poverty population is highly exposed to the effects of climate change; in fact, already in medium-term scenarios the impacts on the fuel poverty population take on Medium High and High values over more than 50% of the municipal extension; this value exceeds 80% in the long-term scenario.

#### Portability test: residential bulding vulnerability on municipality of Avellino (Italy)

**Figure 13:**
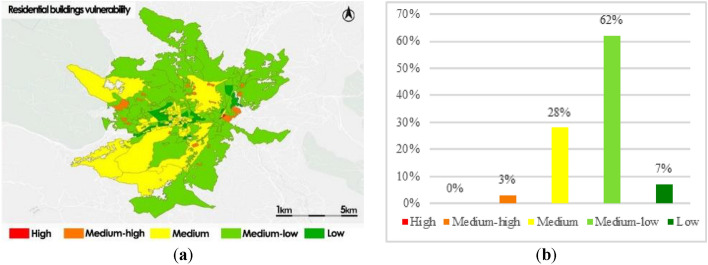
Buildings subsystem vulnerability of Avellino (**a**); frequency histogram (**b**).

In order to evaluate the portability of our framework, we’ve tested it in the study area of the municipality of Avellino. As a demonstration, we show the findings obtained from the assessment of the building system heatwave vulnerability. As evidence of this, the results of the experimentation in the form of thematic maps are represented in Fig. 13.

The thematic map in Fig. 13a shows a prevailing condition of Medium–low vulnerability throughout the municipality (62%). In particular, the census sections in the city center areas are mainly classifies with Low vulnerability and the census sections in the surrounding areas, are mainly classified whit a medium–low vulnerability. The census section classified with Medium or Medium–high vulnerability are those with the highest built and population densities.

As can be seen from the histogram (Fig. 13b), overall, sections classified with Low and Medium–low residential buildings vulnerability prevail (69%); 28% of sections are classified with Medium vulnerability and only 3% are classified with Medium–high vulnerability. This distribution of the residential building heatwave vulnerability by census zone in the municipality of Avellino is consistent with the type of urban pattern characterized by a low population density, perfectly, in contrast to the city of Naples where the population density is much higher.

The fact that the results obtained are in line with the typology of the two urban patterns demonstrates the adaptability of our framework to model the assessment of heatwave vulnerability and impact scenarios of different urban fabrics.

In Table 10, for each vulnerability class, the mean value of the four intermediate indicators is shown:

**Table 10:** Mean class value of the four Residential building intermediate indicators, computed for each vulnerability class.

Vulnerability class	Building volume	Thermal lag	Thermal decrement factor	Solar exposure
Medium–high	Medium–high	High	High	Medium–low
Medium	Medium	Medium–high	Medium–high	Low
Medium–low	Medium–low	Medium	Medium	Low
Low	Medium	Medium–low	Medium–low	Low

Based on Table 4's findings, the vulnerability class is most significantly impacted by three indicators: Solar exposure Thermal lag and Thermal decrement factor, the former remains constant on average as the vulnerability class varies; its value is Medium–low, when the vulnerability class is Medium–high, and it becomes Low, for all other vulnerability classes. Instead, the average values of the Thermal lag and Thermal decrement factor rindicators are High and Medium–high, respectively when the vulnerability class is Medium–high and Medium. This fact highlights that these indicators are the ones that most determine the vulnerability class of residential buildings. In fact, the most vulnerable census areas of Avellino, classified with Medium–high and Medium vulnerability, are those in which a more recent construction in reinforced concrete with more than two blocks is more frequent; in them, the contribution of the vertical closures of buildings prevails in the evaluation of the Thermal lag and Thermal decrement factor indicators.

## Discussion

The test’s results on the urban fabric of Naples (Italy), show that the aggregation by census zone represents a good trade-off between the accuracy of the resulting maps and the portability of the model. In fact, the vulnerability maps of the two subsystems and the resulting impact scenarios show spatial distributions consistent with the various types of urban forms present in the city. The vulnerability analysis exactly reflects the technical-constructive buildings characteristics and the formation of open spaces. From checks carried out with expert decision-makers, it has emerged that the most vulnerable areas are really the most critical in terms of the vulnerability for its morphological and technical-constructive characteristics. This mainly relates to the basic characteristics that were identified for defining the indicators, which are sufficient to assess the urban system's vulnerability. Analyzing the short-, medium-, and long-term impact scenario maps, it is possible to see that the worsening risk is directly proportional to the duration of the heatwave. The census sections classified with Medium–low vulnerability being subjected to a High impact in future scenarios. This implies the need to intervene with mitigation and adaptation solutions in order to prevent the deterioration of the conditions of the urban system components.

A crucial decision that needs to be made in order to model the entire study area reflecting the conditions of the constituent elements while maintaining an average detail scale, is the use of census sections as the spatial atomic information for the maps. The use of a higher detail scale, in which each single building or open space is used as an atomic unit, does not guarantee the fundamental characteristic of portability necessary to replicate the analysis on different urban fabrics, due to the difficulties of finding certified data at that scale of detail. The information sources at this level of detail are often not homogeneous, not limited, not very reliable, or almost out of date.

To evaluate the portability of our framework on other urban fabrics, the GIS-based framework has been used to create the residential buildings heatwave vulnerability maps of the municipality of Avelino (Italy). To produce this map, the same types of spatial datasets used to obtain the Naples thematic map of vulnerability were used, in order to analyze the portability level of the framework by exploring a different urban fabric starting from the same type of source data. This map shows that the most vulnerable census sections (classified with Medium–high and Medium vulnerability) are those of recent construction in which the contribution of the vertical component of the building prevails, which affects the Thermal Lag and Thermal decrement factor indicators. Because of this, even if the urban fabric of Avellino and Naples are very different, the conclusions derived using the framework are still valid. This demonstrates how the suggested GIS-based methodology may be used to analyze heatwave susceptibility and effect scenarios in different urban patterns characterized by specific building fabrics and urban shapes.

## Conclusions

In this paper, we propose a GIS-based framework, developed in the PLANNER research project, for the assessment of vulnerability and impact scenarios to heatwaves in different urban fabrics.

The framework implements the model of heatwave vulnerability and impacts in urban systems proposed in^15^, using the census zone as an atomic spatial entity, which represents the smallest area of the urban fabric with homogeneous urban characteristics; periodic censuses are provided on various characteristics referring to the resident population, residential buildings, families, and buildings. This was done in order to ensure, in addition to the good accuracy of the results, the portability of the framework in different urban fabrics, in which it is always possible to use basic cartographic, satellite data and aggregated census data.

In order to evaluate the spatial distribution of vulnerability and impacts on a study area characterized by a multitude of different urban forms and inhabited by a high population density characterized by different social classes, which represent different types of subjects exposed to risk, it was decided to test the model on a complex urban fabric, such as the city of Naples. Thematic maps of vulnerability and impact scenarios demonstrate that our approach produces findings that are compatible with the geographical distribution of the many building types, urban forms, and social fabrics that are present. As a consequence, it provides a good compromise between the mobility of the model and the findings' correctness.

It was tested for the assessment of the vulnerability of residential buildings in the study area of Avellino (Italy) in order to confirm the portability of the framework in various urban fabrics; the most vulnerable areas are those of new construction, in which the contribution of the vertical component of the building is relevant. This finding indicates how the framework may easily be modified to accommodate urban fabrics with various physical and morphological properties.

To improve the accuracy and portability of the model, we intend to conduct in the future a lot of framework tests on further urban fabrics on an international scale, in order to analyze territorial contexts characterized by different urban forms and social fabrics. In this approach, it is possible to evaluate the real requirement for morphological and technical-constructive characteristics to be added to or modified in connection to the system's components (buildings and open spaces) in order improve the assessments of vulnerability and impacts on the heatwave phenomena.

## Data Availability

The datasets generated during and/or analysed during the current study are available from the corresponding author on reasonable request.
